# Astragaloside IV alleviates inflammation and improves myocardial metabolism in heart failure mice with preserved ejection fraction

**DOI:** 10.3389/fphar.2024.1467132

**Published:** 2024-11-20

**Authors:** Xiao Wang, Xinting Chen, Yuting Wang, Xinyu He, Lan Li, Xiaodan Wang, Yuting Huang, Guanwei Fan, Jingyu Ni

**Affiliations:** ^1^ First Teaching Hospital of Tianjin University of Traditional Chinese Medicine, National Clinical Research Center for Chinese Medicine Acupuncture and Moxibustion, Tianjin, China; ^2^ Tianjin State Key Laboratory of Component-Based Chinese Medicine, Tianjin University of Traditional Chinese Medicine, Tianjin, China; ^3^ Key Laboratory of Prevention and Treatment of Cardiovascular and Cerebrovascular Diseases of Ministry of Education, First Affiliated Hospital of Gannan Medical University, Gannan Medical University, Ganzhou, China

**Keywords:** HFpEF, astragaloside IV, inflammation, metabolism, mitochondria

## Abstract

**Background:**

Heart failure with preserved ejection fraction (HFpEF) has grown to become the dominant form of heart failure worldwide. However, no unequivocally effective treatment for HFpEF has been identified in clinical trials. In this study, we report that Astragaloside IV (AS-IV) can be used to treat HFpEF.

**Methods:**

Mice were fed on a high-fat diet and given 0.5 g/L L-NAME (in drinking water) for 10 weeks to establish the HFpEF model. After 10th weeks, the HFpEF mice were given 10 mg/kg empagliflozin, 10 mg/kg AS-IV, or 20 mg/kg AS-IV for 4 weeks. The echocardiography, blood pressure, hemodynamics, heart failure biomarkers, collagen deposition and fibrosis, histopathology, and inflammation in HFpEF mice were evaluated. Metabolic profiling based on NMR measurements was also performed. Myocardial glucose and fatty acid metabolism were evaluated.

**Results:**

AS-IV improves cardiac function and myocardial remodeling in HFpEF mice. AS-IV attenuates systemic inflammatory infiltration and myocardial inflammation levels in HFpEF mice by decreasing the expression of plasma inflammatory markers GDF15, CRP, IL1RL1, and MCP-1, NLRP3, IL-1β, Caspase-1, and IL-6 in the myocardium of HFpEF mice. Metabolomic analysis suggested that AS-IV improved cardiac glucose and fatty acid metabolism in HFpEF mice. Further studies showed that AS-IV significantly improved Complex I activity, increased ATP production, and elevated plasma NAD + levels; AS-IV also significantly improved pyruvate dehydrogenase activity and decreased pyruvate and lactate accumulation, thereby improving glucose metabolism in the hearts of HFpEF mice.

**Conclusion:**

These results provide novel evidence that Astragaloside IV alleviates inflammation and improves myocardial metabolism in HFpEF mice.

## 1 Introduction

Heart failure has the characteristics of high incidence, high mortality rate, high readmission rate, and high medical expenses. It has become a significant public health problem that seriously affects residents’ health. In the community, approximately 50% of patients with the clinical syndrome of heart failure (HF) have a preserved ejection fraction (HFpEF) (Dunlay et al., 2017). HFpEF has grown to become the dominant form of heart failure worldwide, in tandem with the aging of the general population and the increasing prevalence of obesity, diabetes mellitus, and hypertension (Borlaug, 2020; Omote et al., 2022). Although cardiovascular mortality in HFpEF is lower when compared to HF with reduced ejection fraction (HFrEF), hospital readmissions are frequent, and quality of life is poor (Reddy et al., 2020). Moreover, no unequivocally effective treatment for HFpEF has been identified in clinical trials (Omote et al., 2022; Reddy et al., 2020). Therefore, more in-depth exploration of the occurrence and development mechanism of HFpEF and the development of new treatment strategies are still needed at this stage.

Cardiac mitochondrial function and metabolism are abnormal in the hearts of patients with HFpEF. Patients with HFpEF have resting ATP levels of 20%–40% that of healthy individuals, and their supply of ATP is further reduced due to impaired glycolipid metabolism (Mishra and Kass, 2021). Metabolic disturbance contributes significantly to HFpEF pathogenesis (Schiattarella et al., 2021). Commonly coexisting with other metabolic diseases, such as obesity, diabetes, and hypertension, HFpEF is considered the cardiac manifestation of a systemic metabolic disturbance (Tong et al., 2021). Mitochondrial dysfunction is involved in the pathogenesis of the above comorbidities, so improving mitochondrial function to prevent and treat HFpEF has become a therapeutic method worth exploring.

The main component of Radix Astragali in the treatment of HF is Astragaloside IV (AS-IV). Multiple evidence supports the efficacy and safety of AS-IV *in vivo* and *in vitro* HF models (Zang et al., 2020). AS-IV could protect myocardial ischemia, regulate sarcoplasmic reticulum Ca2+ pump, promote angiogenesis, improve energy metabolism, inhibit cardiac hypertrophy and fibrosis, reduce myocardial cell apoptosis, etc., which are directly or indirectly involved in the beneficial effects of AS-IV in rodents or cellular models of HF (Zang et al., 2020). However, it is currently unclear whether astragaloside IV has any effect on HFpEF and the mechanism by which it exerts its pharmacological effects.

Schiattarella and his colleagues reported a two-hit mouse model of HFpEF with symptoms of obesity and glucose intolerance caused by a high-fat diet (60% of calories from fat) and hypertension caused by Nω-nitro-L-arginine methyl ester (L-NAME) (Schiattarella et al., 2019). Although this model cannot replicate all the pathogenic factors of HFpEF, it combines two major risk factors: metabolic abnormalities and hypertension, characterized by impaired cardiac filling, exercise intolerance, pulmonary congestion, and inflammation (McHugh et al., 2019). Therefore, this method was chosen for the establishment of the HFpEF model. In this experiment, it was revealed whether AS-IV can improve left ventricular dysfunction in HFpEF.

## 2 Materials and methods

### 2.1 Animals and treatment

Male C57BL/6N mice (8 weeks) were from Beijing Charles River Animal Co., Ltd. (Beijing, China). L-NAME was supplied in the drinking water for the indicated periods of time, after adjusting the pH to 7.4. Mice were fed on an HFD (D12492) and given 0.5 g/l L-NAME (in drinking water) for 10 weeks to establish the HFpEF model (Schiattarella et al., 2019). The standard controls (control group) were fed on control feed and ordinary drinking water. After 10th weeks, the HFpEF mice were given 10 mg/kg empagliflozin (oral gavage), 10 mg/kg AS-IV (intraperitoneal injection), or 20 mg/kg AS-IV (intraperitoneal injection) for 4 weeks.

### 2.2 Blood pressure recordings

The tail-cuff method and an animal non-invasive blood pressure monitor (BP98AWU, Tokyo, Japan) were used to evaluate blood pressure in conscious mice. The mice were placed in a cylindrical heating sleeve and heated at 37°C for 3 min. The non-invasive blood pressure meter was used to measure the blood pressure of the mice under conscious conditions and in a quiet environment until three stable continuous measurements, including SBP and DBP, were obtained.

### 2.3 Echocardiography

A Vevo2100 ultrahigh-resolution ultrasound system (VisualSonics, Canada) was used for standard transthoracic echocardiogram analysis after 28 days of drug intervention. The left ventricular parameters, including ejection fraction (EF), fractional shortening (FS), interventricular septum thickness (IVS), LV posterior wall thickness (LVPW), and LV mass, were measured via Standard B-mode and M-mode imaging. The isovolumic relaxation time (IVRT) and isovolumic contraction time (IVCT) were evaluated using a pulse wave Doppler.

### 2.4 Hemodynamic measurement

The cardiac hemodynamics were monitored using the Millar pressure-volume system. The catheter was connected to a microcomputer instrument via a pressure transducer and then inserted into a 5 mL syringe filled with room-temperature PBS buffer for calibration. The anesthetized mouse, injected with 0.1 mL of bromoethanol per 20 g, was fixed on a mouse board. A longitudinal incision of about 2 cm was made from the neck, and the muscle was bluntly separated using toothless microforceps to expose the trachea. A retention needle was used for tracheal intubation. The ventilator was connected, the respiratory rate was adjusted, the chest skin was cut, the muscle was bluntly separated, and the hemostatic forceps were used to separate the third and fourth intercostal spaces bluntly. The pericardium was torn open, and the heart was extruded. The catheter was inserted into the left ventricle from the apex. After the waveform stabilized, the left ventricular systolic pressure (Pes), left ventricular diastolic pressure (Ped), left ventricular maximum rate of rise and fall (±dp/dt max), and minimum rise/fall rate (dp/dt min) were recorded.

### 2.5 Histological analysis

The heart tissues were fixed using 4% paraformaldehyde for 48 h, subsequently embedded in paraffin, and sliced (4–5 μm). Histologic sections of tissues were stained with hematoxylin/eosin (H&E), masson, sirius red, and wheat germ agglutinin (WGA).

### 2.6 Exercise tolerance tests

Firstly, the mice were subjected to adaptive training on a treadmill with a slope of 0°, a speed of 9 m/min, a current of 0.25 mA, and trained for 30 min. The formal experiment was conducted on the fourth day. The experimental conditions are a slope of 20°, with a speed of 9 m/min for 5 min to adapt, and then the speed is increased to 12 m/min, with a speed increase of 1 m/min every 10 min and a current of 0.25 mA. The endpoint is determined to be that the mouse cannot maintain normal running speed and state after receiving a continuous 3-s electric shock. The mouse is removed from the track and the running distance is recorded.

### 2.7 Mitochondrial ultrastructure

For TEM, heart tissues were processed for ultrastructural analysis using the method previously described (Li et al., 2020; Kirshenbaum and Singal, 1992). Left ventricles were cut into 1 mm^3^ random areas of the cubes. Heart cubes were fixed in 2% glutaraldehyde. Then, they were osmicated in 2% OsO4, after which ultrathin sections were stained with uranyl acetate and lead citrate and then subjected to ultrastructural examination. Pictures were taken from three random areas from three sections per mouse.

### 2.8 Metabolomics analysis

The mice were euthanized in the morning for metabolomics analysis. Metabolic profiling based on NMR measurements was performed using the method previously described (Jiang et al., 2019). The raw data from the mass spectrometer downstream were converted to mzML format by ProteoWizard, and peak extraction and retention time correction were performed using the XCMS program. Peak areas were corrected using the “SVR” method, and peaks with >50% missing were filtered in each sample group. The corrected and filtered peaks were searched in the laboratory’s database and integrated with public libraries, AI prediction libraries, and metDNA methods to obtain metabolite identification information.

### 2.9 Quantitative real-time PCR (qRT-PCR)

Real-time quantitative PCR was performed as previously described (Kirshenbaum and Singal, 1992). The primer sequences of ANP, BNP, a-SKA, and b- MHC were synthesized by Sangon Biotech Co., Ltd. (Shanghai, China) as follows: for ANP, AAG​AAC​CTG​CTA​GAC​CAC​CTG​GAG and TGC​TTC​CTC​AGT​CTG​CTC​ACT​CAG; for BNP, TAA​CGC​ACT​GAA​GTT​GTT​GTA​GG and CGC​TAT​GTT​TAT​TAT​GTT​GTG​GC; for Collagen I, GAC​AGG​CGA​ACA​AGG​TGA​CAG​AG and CAG​GAG​AAC​CAG​GAG​AAC​CAG​GAG; for Collagen III, ACG​AGG​TGA​CAA​AGG​TGA​AAC​TGG and AGA​ACC​TGG​AGG​ACC​TGG​ATT​GC; for CTGF, CAA​AGC​AGC​TGC​AAA​TAC​CA and GGC​CAA​ATG​TGT​CTT​CCA​GT; for IL-1β, TCG​CAG​CAG​CAC​ATC​AAC​AAG​AG and AGG​TCC​ACG​GGA​AAG​ACA​CAG​G; for IL-6, CTT​CTT​GGG​ACT​GAT​GCT​GGT​GAC and AGG​TCT​GTT​GGG​AGT​GGT​ATC​CTC; for Caspase-1, ACA​ACC​ACT​CGT​ACA​CGT​CTT​GC and CCA​GAT​CCT​CCA​GCA​GCA​ACT​TC; and for GAPDH, CGGCCGCATCTTCTTGTG and CACC GACCTTCACCATTTTGT.

### 2.10 Western blot analysis

Western blot analysis was performed using the method previously described (Ni et al., 2020). The heart protein was extracted with lysis buffer (Solarbio, Beijing, China). Lysates were centrifuged, and supernatants were collected. The protein content of the lysates was quantified using a BCA assay kit (Thermo Fisher Scientific, United States). Protein samples were resolved by SDS-PAGE and transferred to Polyvinylidene fluoride membranes (Millipore, Bedford, MA, United States) in a wet transfer system (Bio-Rad, Hercules, CA, United States). Membranes were blocked with 5% defatted milk powder for 2 h at room temperature and subsequently immunoblotted with specific primary antibodies against MMP1, MMP2, MCP1, NLRP3, VCAM1, MPC1, GLUT1, GLUT4, Ndufb8, SDHB, UQCRC2, MTCO2, ATP5A1, and GAPDH at 1,000 times dulition (Proteintech or CST, United States) overnight. Then the membrane was incubated at room temperature for 2 h with horseradish peroxidase-conjugated secondary antibody diluted 10,000 times (Proteintech, China). Specific protein bands were visualized by enhanced chemiluminescence (Beyotime, Shanghai, China) and quantified by densitometry with an Amersham Imager 680 ultra sensitive chemiluminescence imaging instrument (GE Healthcare, United States). The relative values were corrected to GAPDH expression levels and normalized with respect to baseline controls.

### 2.11 Measurement of PDH activity, LDH activity, and ComplexⅠ activity

PDH activity and LDH activity were examined with commercial kits (Solarbio, Beijing, China). ComplexⅠ activity was examined with commercial kits (Abbkine Scientific Co., Ltd., Wuhan, China). Hearts from mice fasted overnight were homogenized in PBS. PDH activity, LDH activity, and ComplexⅠ activity were determined as above.

### 2.12 Statistical analysis

The experimental processes and data analyses were carried out following randomization. Statistical analysis was performed by one-way ANOVA or Kruskal–Wallis test of repeated experiments followed by SPSS 22.0 statistical software. A *p*-value <0.05 was considered statistically significant.

## 3 Results

### 3.1 Astragaloside IV improves cardiac function in HFpEF mice

**FIGURE 1:**
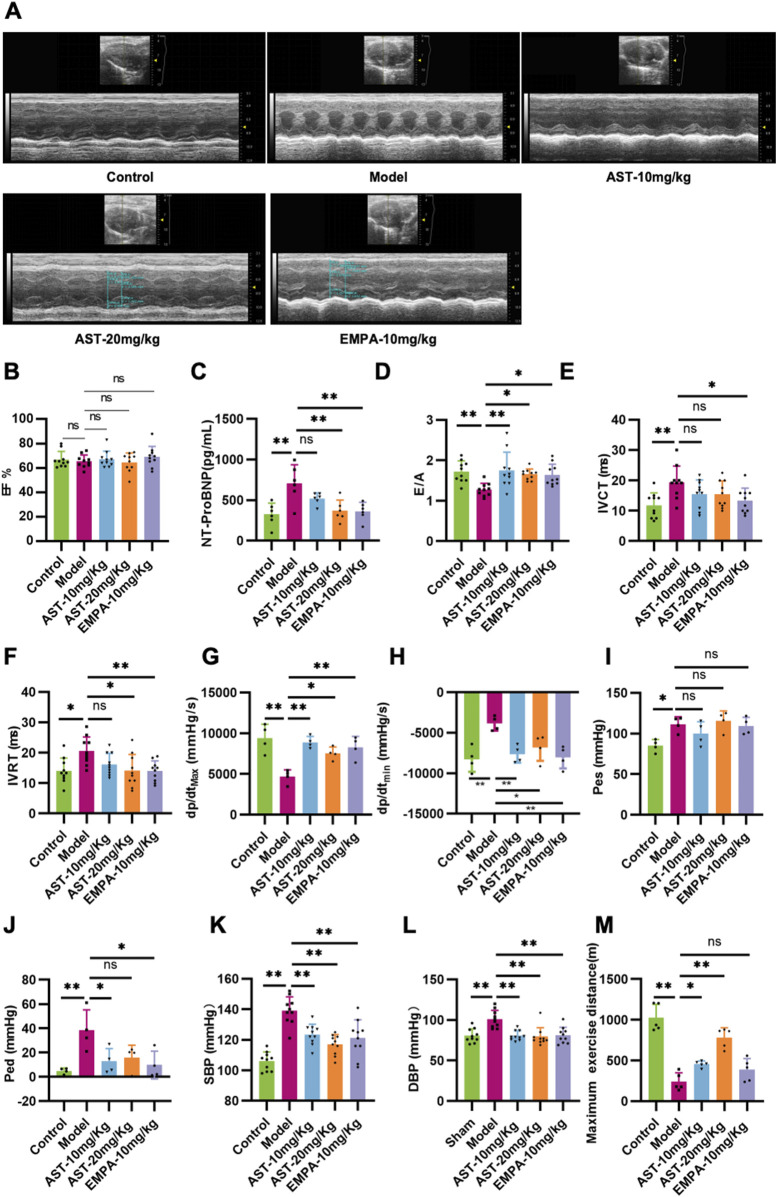
Astragaloside IV improves cardiac function in HFpEF mice. **(A)** A long-axis view of the heart in the M-type echocardiogram. **(B)** Left ventricular ejection fraction (EF). **(C)** The content of the Amino-terminal pro-brain natriuretic peptide (NT-proBNP) in plasma was determined by ELISA. **(D)** Mitral inflow E and A wave ratio (E/A). **(E)** Isovolumic contraction time (IVCT). **(F)** Isovolumic relaxation time (IVRT). **(G)** Maximum rise/fall rate (dp/dt max). **(H)** Minimum rise/fall rate (dp/dt min). **(I)** Left ventricular end-systolic pressure (Pes). **(J)** Left ventricular end-diastolic pressure (Ped). **(K)** Systolic blood pressure (SBP). **(L)** Diastolic blood pressure (DBP). **(M)** Running distance during exercise exhaustion test.

To investigate the effect of AS-IV in HFpEF, either AS-IV (10 mg/kg/day or 20 mg/kg/day) or saline was administered daily via intraperitoneal injection. After 4 weeks of treatment, echocardiography was performed. The transverse echocardiographic evaluation showed that the EF was unchanged in all groups (Figures 1A, B). The content of NT-proBNP, a heart failure marker, significantly increased in the plasma of HFpEF mice. After treatment with AS-IV, the content of NT-proBNP was significantly reduced (Figure 1C). The pulse Doppler results showed that the E/A value of HFpEF mice was significantly reduced, and both IVCT and IVRT were prolonged. After intervention with astragaloside IV, the E/A value was significantly increased, and both IVCT and IVRT were shortened (Figures 1D–F). The LV compliance and hemodynamics were further evaluated via invasive cardiac catheterization. The maximum rise/fall rate (dp/dt max) and minimum rise/fall rate (dp/dt min) significantly decreased in HFpEF mice, indicating a loss of LV compliance (Figures 1G, H). Moreover, the reduced LV compliance was further confirmed by the increased LV pressure (Pes and Ped) in HFpEF mice (Figures 1I, J). It was found that AS-IV significantly increased dp/dt max and dp/dt min, and reduced Ped (Figures 1G–J). Furthermore, it was found that HFpEF mice showed significant hypertension compared to healthy controls. This was reflected by the significantly higher blood pressure (both systolic and diastolic) in HFpEF mice (Figures 1K, L). By contrast, systolic and diastolic blood pressure (SBP and DBP) significantly decreased after AS-IV treatment, indicating that AS-IV can prevent hypertension in HFpEF mice. Through exercise tolerance testing, it can also be seen that the motor function of HFpEF mice significantly declined, and the administration of AS-IV improved these conditions (Figure 1M). These results indicated that AS-IV relieved HFpEF cardiac function.

### 3.2 Astragaloside IV improves myocardial remodeling in HFpEF mice

**FIGURE 2:**
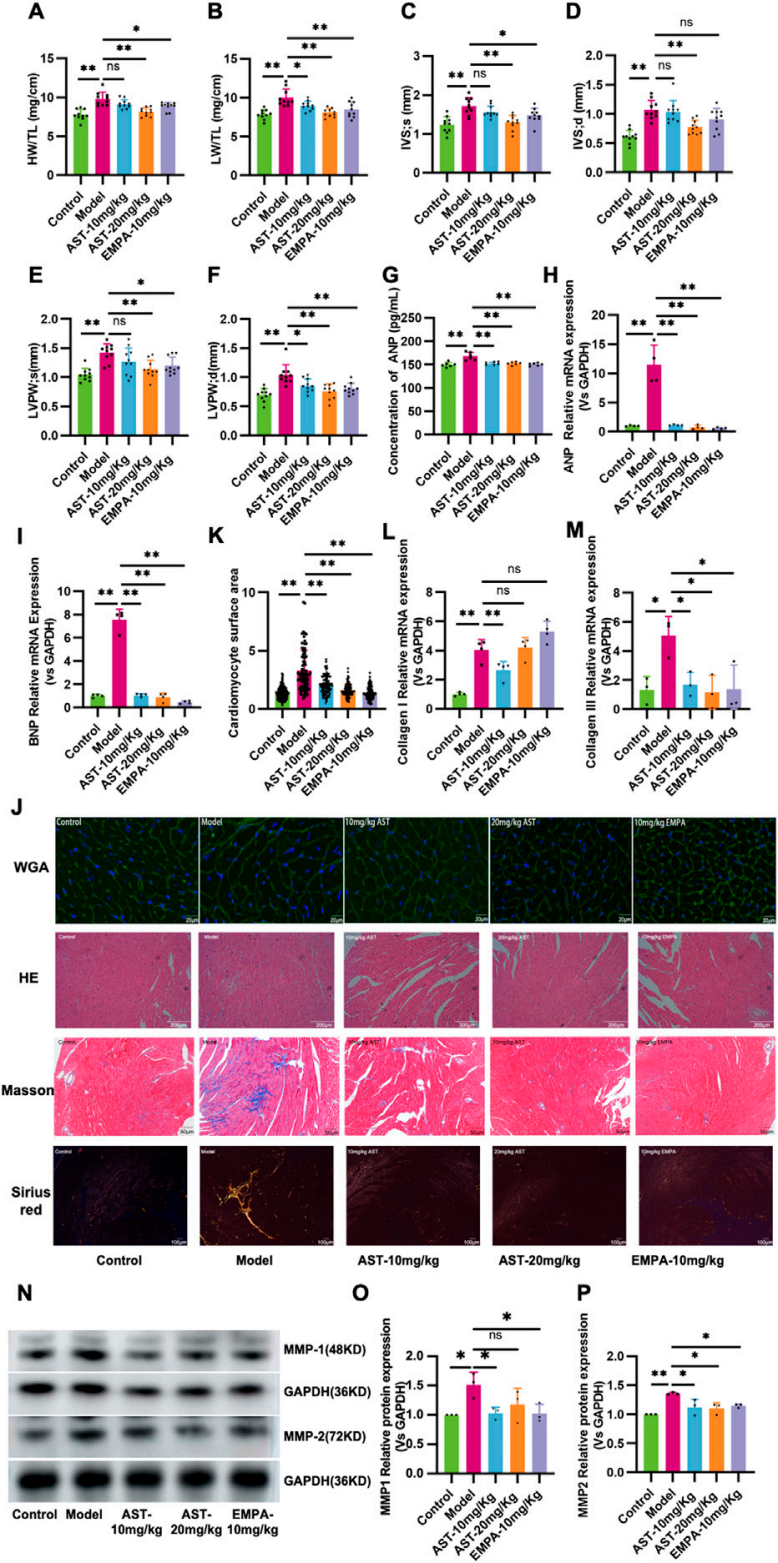
Astragaloside IV improves myocardial remodeling in HFpEF mice. **(A)** Heart weight/tibia length ratio (HW/TL). **(B)** Left ventricular mass (LV Mass). **(C)** Systolic interventricular septal thickness (IVS;s). **(D)** Diastolic interventricular septal thickness (IVS;d). **(E)** Systolic left ventricular posterior wall thickness (LVPW;s). **(F)** Diastolic left ventricular posterior wall thickness (LVPW;d). **(G)** The atrial natriuretic peptide (ANP) content in plasma is determined by ELISA. **(H,I)** ANP and Brain natriuretic peptide (BNP) mRNA expression. **(J,K)** Representative images of wheat germ agglutinin (WGA) staining and the calculated myocyte cross-sectional area. **(J)** Representative myocardial histological images of HE staining, Masson staining, and Sirius red staining. **(L,M)** Collagen I and Collagen III mRNA expression. **(N-P)** Western blot analysis of matrix metalloproteinase 1 and 2 (MMP-1, MMP-2) in the myocardium.

HFpEF mice showed pathological hypertrophy of the entire myocardium, as reflected by increased HW/TL, LV Mass/TL, IVS, and LVPW (Figures 2A–F). Plasma ANP levels significantly increased in HFpEF mice (Figure 2G). Furthermore, myocardial ANP and BNP mRNA levels significantly increased in HFpEF mice (Figures 2H, I). These heart function parameters were significantly reduced after 28 days of AS-IV treatment, indicating that AS-IV relieves HFpEF myocardial hypertrophy. WGA showed cardiomyocyte hypertrophy in HFpEF mice (Figures 2J, K). The differences between cardiomyocytes and their surface area were significantly increased. AS-IV treatments reduced the cardiomyocyte difference and significantly reduced the surface area of cardiomyocytes (Figures 2J, K). HE staining (Figure 2J) showed nucleus aggregation and disordered cell arrangement in HFpEF mice. AS-IV treatment group showed different relief in myocardial necrosis after 28 days of treatment. Masson staining and Sirius red staining showed significant collagen deposition and fibrosis in the myocardial tissue of HFpEF mice, and administration of astragaloside significantly reduced collagen deposition and fibrosis in HFpEF myocardium (Figure 2J). Moreover, AS-IV treatment also reduced the collagen area and the expression levels of collagen I and III (Figures 2L, M). Furthermore, cardiac matrix metalloproteinase protein levels (MMP-1 and MMP-2) were significantly restored in the AS-IV-treated mice compared with the model mice (Figures 2N–P).

### 3.3 Astragaloside IV inhibits the activation of systemic and myocardial inflammation in HFpEF mice

**FIGURE 3:**
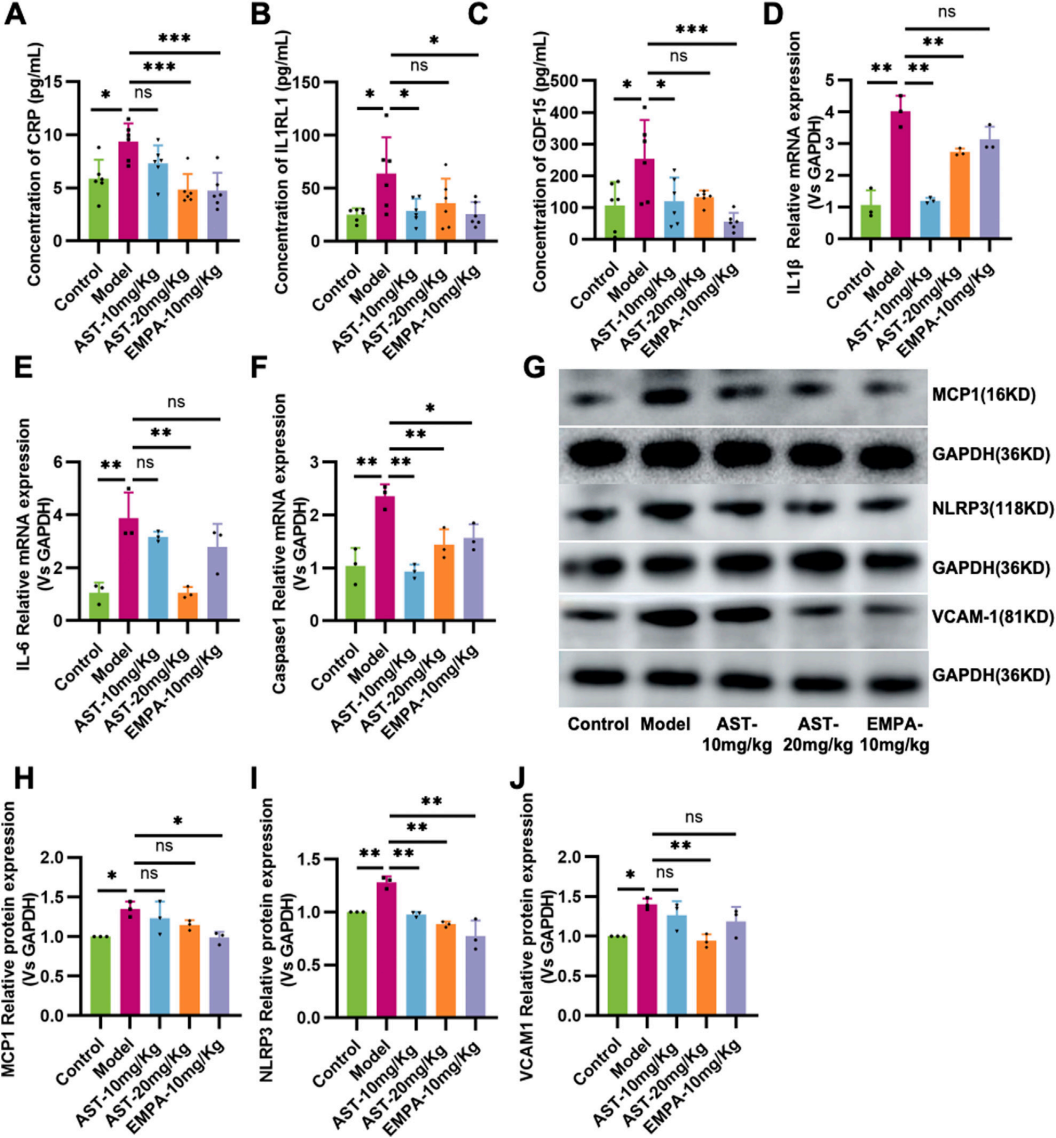
Astragaloside IV inhibits inflammation in HFpEF mice. **(A)** Plasma C-reaction protein (CRP). **(B)** Plasma Recombinant Interleukin-1 Receptor Like-1 (IL1RL1) content. **(C)** Plasma Growth and Differentiation Factor 15 (GDF15) content. **(D–F)** The content of IL-1β, IL-6, and Caspase-1 in the myocardium. **(G–J)** Western blot analysis of MCP-1, NLRP3, and VCAM-1 in the myocardium.

The expression of inflammatory factors CRP, IL1RL1, and GDF15 increased in the heart of HFpEF mice, suggesting that HFpEF is associated with the activation of Systemic inflammation (Figures 3A–C). AS-IV treatment reduced CRP, IL1RL1, and GDF15 levels, indicating a slightly decreased systemic inflammatory status. HFpEF mice showed increased mRNA levels of IL-1, IL-6, and Caspase-1 in the myocardium. Western blot results showed that MCP-1, NLRP3, and VCAM-1 expression in HFpEF mice was significantly upregulated. These genes promote the production of pro-inflammatory cytokines. After administration of astragaloside IV, the expression of IL-1, IL-6, Caspase-1, NLRP3, and VCAM-1 in the myocardium of HFpEF mice was significantly reduced (Figures 3D–J).

### 3.4 Astragaloside IV mediates abnormalities in energy metabolic pathways at the metabolite levels in HFpEF mice

**FIGURE 4:**
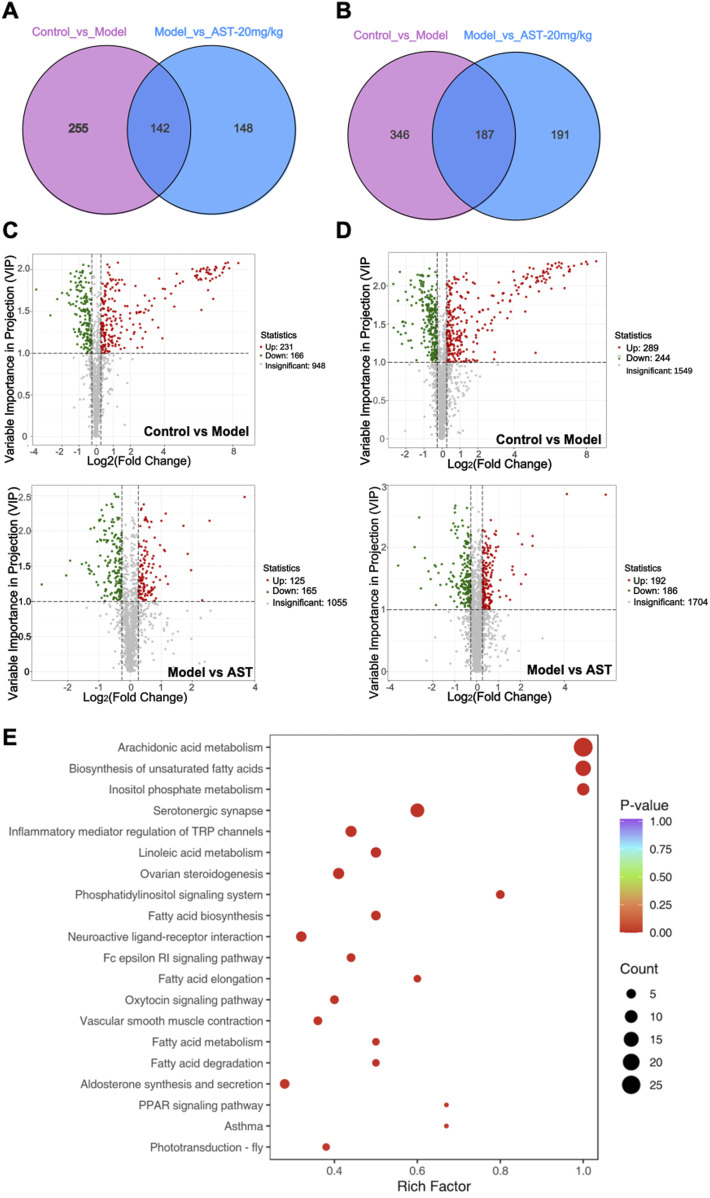
Metabolomics results. **(A, B)** Venn diagrams showing the number of differentially expressed metabolites (DEMs) in HFpEF model mouse versus sham mouse and AS-IV-treated HFpEF mouse versus models. **(C, D)** Volcano plot showing DEM distribution in HFpEF model mouse versus control mouse and AS-IV-treated HFpEF mouse versus models. **(E)** KEGG pathway enrichment analysis of DEMs.

A total of 1,598 metabolites were identified as differentially expressed metabolites (DEMs) at the metabolome level, out of which 930 were altered in HFpEF mice relative to WT mice, while 668 DEMs were restored through AS-IV treatment (Figures 4A–E). Cluster analysis showed significant changes in metabolites between different groups. The volcano and VENN plots showed that 410 differential metabolites were significantly downregulated, and 520 differential metabolites were significantly upregulated in HFpEF mice. After treatment with astragaloside IV, 351 differential metabolites were significantly downregulated, and 317 differential metabolites were significantly upregulated (Figures 4A–D). Pathway enrichment analysis based on the KEGG database was performed to evaluate the potential function of DEMs. As shown in Figure 4E, DEMs were significantly enriched in pathways related to energy metabolism, such as Arachidonic acid metabolism, Biosynthesis of unsaturated fatty acids, Inositol phosphate metabolism, TCA cycle, fatty acid biosynthesis, fatty acid elongation, and pyruvate metabolism. This suggested that AS-IV had a significant regulatory effect on energy metabolism in HFpEF mice.

### 3.5 Validation of the effect of astragaloside IV on glucose and fatty acid metabolism in HFpEF mice

**FIGURE 5:**
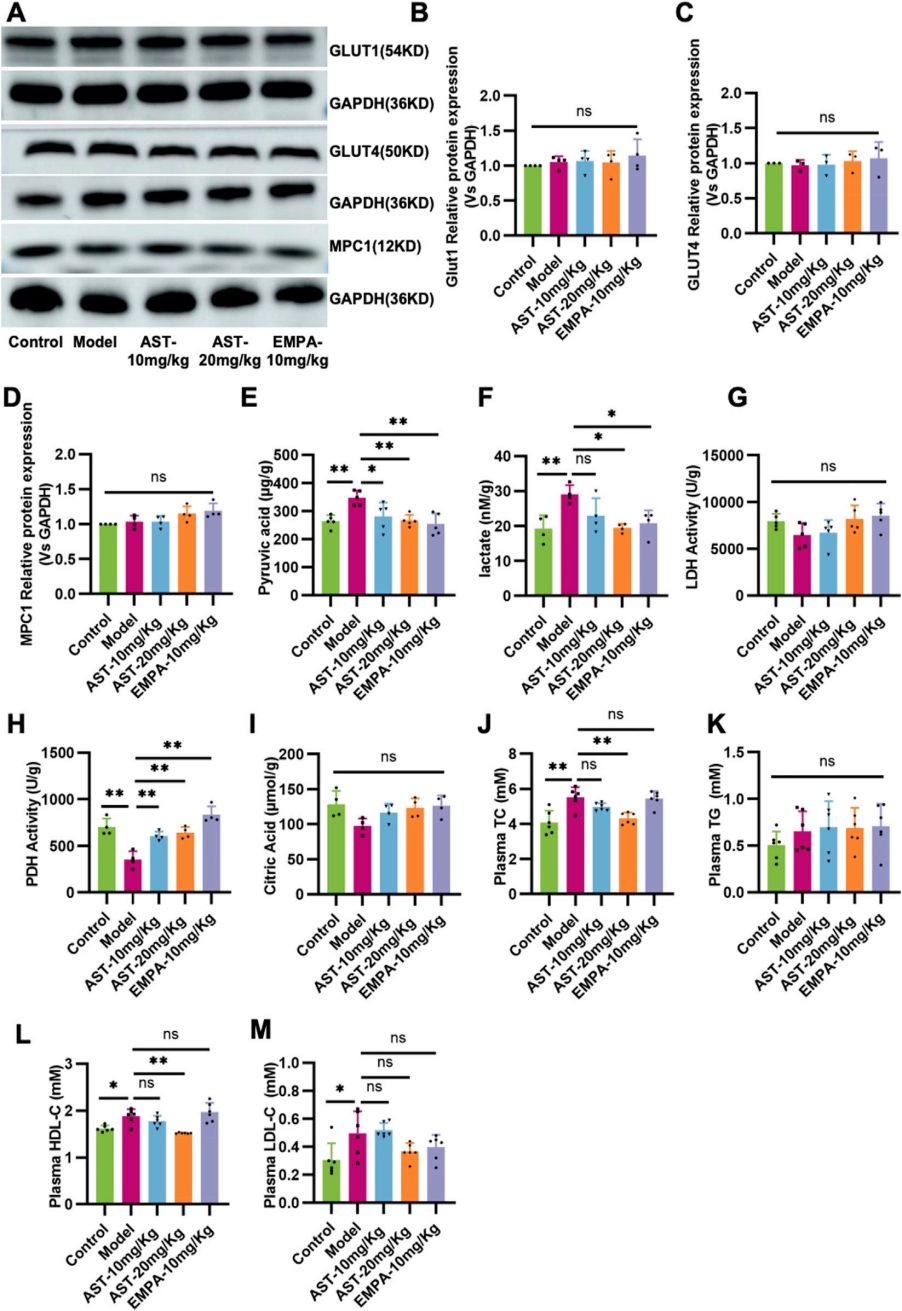
Validation of the effect of AS-IV on glucose and fatty acid metabolism in HFpEF mice. **(A–D)** Western blot analysis of the expression of glucose transporter 1 (GLUT1), glucose transporter 4 (GLUT4), and mitochondrial pyruvate carrier protein 1 (MPC1). **(E)** The contents of pyruvate in the myocardium. **(F)** The contents of lactate in the myocardium. **(G)** The activity of lactic dehydrogenase (LDH) in the myocardium. **(H)** The activity of pyruvate dehydrogenase in the myocardium. **(I)** The contents of citric acid in the myocardium. **(J)** Total cholesterol (TC) levels in plasma. **(K)** Triglyceride (TG) levels in plasma. **(L)** The high-density lipoprotein cholesterol (HDL-C) content in plasma. **(M)** low-density lipoprotein cholesterol (LDL-C) content in plasma.

The vital metabolic enzymes related to glucose and fatty acid metabolism were measured to establish the potential effects of AS-IV in the energy remodeling of HFpEF. As shown in Figures 5A–D, the expression of glucose transporter 1 (GLUT1), glucose transporter 4 (GLUT4), and mitochondrial pyruvate carrier protein 1 (MPC1) remained unchanged in HFpEF mice. The content of pyruvate and lactate, critical metabolites in the glycolysis process, was detected (Figures 5E, F). The results showed that HFpEF mice had lactate accumulation in the myocardium, and there was no significant change in lactate dehydrogenase activity, which mediates lactate production from pyruvate (Figure 5G). However, pyruvate dehydrogenase activity was significantly reduced (Figure 5H). The results showed that in HFpEF mice, the decreased pyruvate dehydrogenase activity resulted in lactate accumulation in myocardial tissue. The content of citric acid during the TCA cycle was detected. However, the model group showed a decrease compared to the normal group and an increase in citric acid content after administration; there was no significant difference (Figure 5I). After administration of AS-IV, it significantly improved the myocardial PDH activity in HFpEF mice, thereby improving the accumulation of pyruvate and lactate in myocardial tissue. Metabolites related to lipid metabolism are detected. The results showed that the levels of total cholesterol (TC), high-density lipoprotein cholesterol (HDL-C), and low-density lipoprotein cholesterol (LDL-C) in the plasma of HFpEF mice were significantly increased (Figures 5J–M). After administration of AS-IV, the content of TC and HDL-C in the myocardium of HFpEF mice was significantly reduced (Figures 5J–M).

### 3.6 Astragaloside IV maintains mitochondrial structure and function in HFpEF mice

**FIGURE 6:**
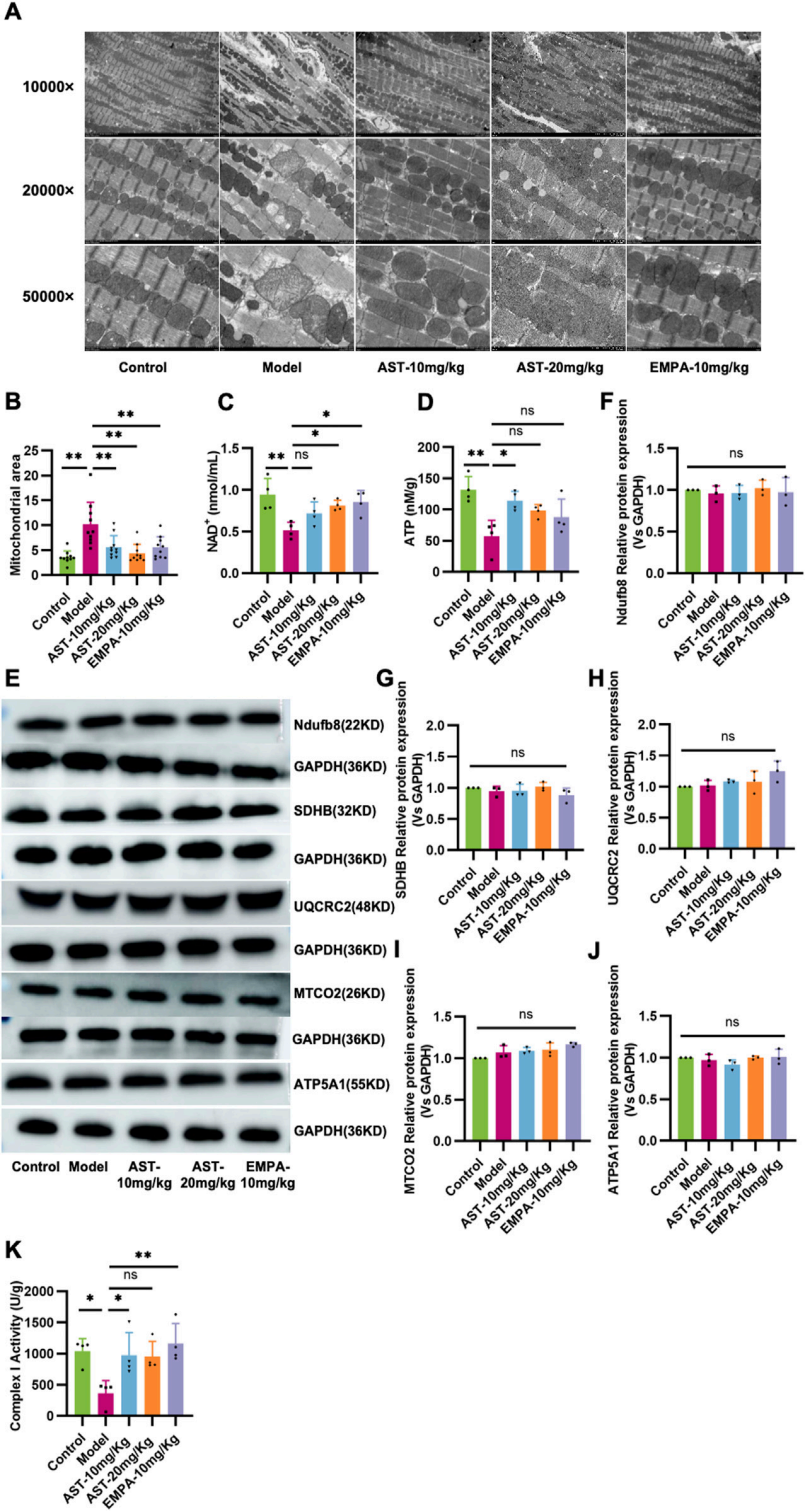
Astragaloside IV maintains mitochondrial structure and function in HFpEF mice. **(A, B)** Representative transmission electron microscopy images showing the mitochondrial network in myocardial tissue in all experimental groups. **(C)** The level of NAD+ in plasma. **(D)** The ATP content in the myocardium. **(E–J)** Western blot analysis was performed to determine expression of mitochondrial complex I (NADH dehydrogenase [ubiquinone] 1 beta subcomplex subunit 8, NDUFB8), complex II (succinate dehydrogenase subunit B, SDHB), complex III (ubiquinol-cytochrome c reductase core protein II, UQCRC2), complex IV (cytochrome C oxidase subunit II, MT-CO2), and complex V (ATP synthase subunit alpha 1, ATP5A1). **(K)** The activity of Complex I in the myocardium.

Ultrastructural aspects of the mitochondria in myocardium cells revealed structural disorders with vacuolar degeneration and edematous myocardial fibers in HFpEF mice (Figures 6A, B). Upon treatment with AS-IV, the mitochondria were neatly arranged, there was a reduction in the mitochondrial edema, and the mitochondria structure became relatively clear and intact. HFpEF mice showed a significant decline in NAD+ and ATP contents compared to control mice (Figures 6C, D); these abnormalities were restored by AS-IV treatment. To further investigate the effect of AS-IV on mitochondrial function, the expression level and activity of the complex protein of the mitochondrial electron transfer chain were detected (Figures 6E–J), and the results showed that there was no significant difference in the protein expression of the mitochondrial electron transfer chain complex among the experimental groups (Figures 6E–I). However, the activity of Complex I was significantly reduced in the model group and improved after administration of AS-IV (Figure 6K). These results indicated that AS-IV’s anti-HF effect regulated mitochondrial function and structure and alleviated mitochondrial energy metabolism dysfunction.

## 4 Discussion

The “2-hit” model is a model of HFpEF induced in C57BL/6N mice by administration of Nω-nitro-L-arginine methyl ester (L-NAME) in drinking water in combination with a high-fat diet. This model combines the multifactorial aspects of hypertension, obesity, and glucose intolerance, and exhibits left ventricular centripetal hypertrophy, pulmonary edema, and reduced exercise capacity. In this experiment, AS-IV was selected as the research object and the “2-hit” HFpEF mouse model was used to explain the medicinal value of AS-IV in HFpEF by combining various experimental methods. The results showed that AS-IV improved the symptoms of HFpEF mice mainly in improving myocardial hypertrophy, pulmonary congestion, reducing SBP, DBP and ANP, NT-proBNP, and obesity and dyslipidemia. Hemodynamic experiments showed that AS-IV increased dp/dtmax, dp/dtmin, and decreased ped in the hearts of HFpEF mice, thus improving cardiac compliance. Decreased exercise tolerance is a significant characteristic of HFpEF patients, which seriously affects the life quality of HFpEF patients. AS-IV significantly improved the maximal exercise distance of HFpEF mice in this study.

AS-IV also improves cardiac function in HFpEF mice, which is consistent with previous studies on the protective effects of astragaloside on cardiac function (Wang et al., 2020). Myocardial remodeling occurred in the hearts of HFpEF mice, which showed myocardial fibrosis and hypertrophy. The improvement of myocardial remodeling by AS-IV is mainly reflected in the decreased expression of collagen I, III, and MMPs. MMPs are protein hydrolases responsible for remodeling and degrading cytoplasmic matrix. While MMP2 is proposed as a HFpEF marker, AS-IV appears more effective in reducing collagen III than collagen I. In future research, we will consider investigating other MMPs, such as MMP1, MMP3, and MMP9, to gain a more comprehensive understanding. Tissue inhibitors of metalloproteinases (TIMPs) are endogenous protease inhibitors that bind to MMPs in a 1:1 ratio to form complexes, regulating the activation or function of MMPs. This study did not investigate the effects of AS-IV on TIMPs. In future research, we will add more indicators to support the research results. AS-IV improved cardiac hypertrophy as WGA staining and ultrasound results showed that astragaloside significantly reduced myocardial surface area and IVS and LVPW in HFpEF mice. L-NAME mainly induced hypertension, while HFD caused lipid dysfunction and metabolic stress in HFpEF mice. In the present study, we found that AS-IV reduced body weight gain and significantly decreased plasma levels of TC and HDLC in HFpEF mice, suggesting that AS-IV modulates lipid dysfunction. These results indicate that cardiac function was significantly improved, and myocardial remodeling was reversed in HFpEF mice after AS-IV treatment, which confirms the therapeutic value of AS-IV in HFpEF.

The cardiac structural and functional alterations of HFpEF are driven by induced systemic inflammation (Paulus and Tschöpe, 2013). Various plasma and myocardial tissue inflammatory markers have been used to diagnose and validate HFpEF. CRP is an independent predictor of mortality in patients with HFpEF (Westermann et al., 2011; Hahn et al., 2020; Chirinos et al., 2020). High levels of CRP are associated with the severity of HFpEF comorbidities and mitochondrial dysfunction (Pellicori et al., 2020; Sanders-van Wijk et al., 2015). IL1RL1 is a peptide belonging to the interleukin-1 receptor family and is secreted by cardiomyocytes and cardiac fibroblasts. Therefore, it is regarded as a biomarker for myocardial fibrosis, cardiac stretching, and remodeling. Measurement of IL1RL1 levels helps to predict the risk of death and prognosis in patients with HFpEF (Cunningham et al., 2020; Rios et al., 2020; Frangogiannis, 2021). GDF15 is associated with exercise capacity, and decreased exercise tolerance is also a significant feature of HFpEF, suggesting that GDF15 has diagnostic value for HFpEF (Cotter et al., 2015). In this study, AS-IV reduced CRP, GDF15, and IL1RL1 plasma levels in HFpEF mice. The results indicated that AS-IV could improve the systemic inflammation in HFpEF, which laterally reflected that it could improve the prognosis of HFpEF.

Clinical studies have shown that inflammatory markers such as IL-1β, IL-6, and IL-10 levels are significantly elevated in the hearts of patients with HFpEF compared to HFrEF (Westermann et al., 2011; Hahn et al., 2020). Increased pro-inflammatory cytokines enhance oxidative stress, drive fibroblast differentiation into myofibroblasts, and induce extracellular matrix degradation, increasing myocardial stiffness and coronary microvascular dysfunction (Paulus and Tschöpe, 2013; Bairey Merz et al., 2020). Myocardial inflammation decreases the bioavailability of nitric oxide and cyclic guanosine monophosphate, leading to increased phosphorylation of myosin, further increasing myocardial stiffness and worsening cardiac function (Paulus and Tschöpe, 2013). In addition, the NLRP3/Caspase-1/IL-1β pathway is present in the inflammatory response in heart disease. After the intervention of AS-IV, the protein expression of Caspase-1, IL-1β, and NLRP3 was significantly reduced. Thus, astragaloside inhibits systemic and myocardial inflammation in HFpEF mice. It is worth noting that AS-IV at 10 mg is more effective in reducing IL-1β and caspase 1 levels. This may be related to the non-linear relationship between the efficacy and dosage of drugs in many cases, that is, within a certain range, as the dosage increases, the efficacy may first increase and then tend to stabilize or decrease. The metabolism of astragaloside IV deserves further investigation. Although caspase 1 levels are reduced after AS-IV treatment, it is the active form of caspase 1 that performs its biological functions in the heart. Assessing caspase 1 activity would provide stronger support for the findings. In future research, we will focus on the importance of caspase-1 activity.

Cardiomyocyte mitochondria in HFpEF have structural and functional abnormalities. This suggests that the pathophysiology of HFpEF involves mitochondrial damage. Mitochondrial structural and functional dysfunction is central to metabolic disorders in cardiomyocytes. Normal mitochondrial morphology is essential for mitochondrial function, calcium cycling, and energy metabolism homeostasis. Mitochondrial function can be assessed regarding mitochondrial morphology, ATP production, and biogenesis in mitochondria (Kumar et al., 2019). In the present study, cardiac metabolic disturbances in HFpEF mice were first identified by metabolomics. Transmission electron microscopy observed that myocardial mitochondria were swollen and had disorganized cristae structures, visualizing the mitochondrial damage. Myocardial ATP content was found to be reduced in HFpEF mice. Although there was no difference in the expression of mitochondrial electron transport chain-related proteins, Complex I activity was reduced. These indices improved after intervention with the administration of AS-IV. Alterations in cardiac energy metabolism are considered an essential factor in the severity of heart failure. However, controversy exists regarding the changes that occur in cardiac energy metabolism in HFpEF.

It has been shown (Fillmore et al., 2018) that the earliest change in cardiac energy metabolism in HFpEF is a dramatic increase in glycolysis. However, despite the increase in glycolysis, pyruvate from glycolysis accumulates in the heart due to inadequate oxygen supply and reduced PDH activity, further exacerbating the increased uncoupling of glycolysis and glucose oxidation. Previous studies have shown that increasing this uncoupling of glycolysis and glucose oxidation leads to intracellular acidosis, which impairs cardiac function (Liu et al., 2002). A compensatory increase in glycolysis caused by a decrease in total mitochondrial oxidative metabolism has also been shown to be associated with diastolic dysfunction (Beer et al., 2002). In the present study, the proteins associated with glucose uptake in myocardial mitochondria did not produce differences. However, pyruvate and lactate accumulation occurred in the heart due to decreased PDH activity. The reduced citric acid content in the myocardium also responded to the reduced flux of metabolic substrates into the TCA cycle. This was improved after AS-IV intervention, demonstrating that astragaloside may exert a protective effect in HFpEF mice by improving mitochondrial function and modulating glucose metabolism. This study has a large proportion of descriptive data, and in future research, we will focus on metabolism to identify a specific pathway that will enhance the impact of the study.

In summary, AS-IV improves cardiac function and structure in HFpEF and its concomitant hypertension, pulmonary congestion, and lipid disorders in the body. AS-IV also reduces systemic and myocardial inflammation in HFpEF, improves mitochondrial function, and improves glucose metabolism disorders in the heart. This proves that AS-IV exerts a protective effect on HFpEF research to provide a basis for clinical application guidance.

## 5 Conclusion

Astragaloside IV exerts cardioprotective effects by attenuating inflammation and improving myocardial metabolism in mice with ejection fraction preserved heart failure.

## Data Availability

The original contributions presented in the study are included in the article/supplementary material, further inquiries can be directed to the corresponding author.

## References

[B1] Bairey Merz C. N. Pepine C. J. Shimokawa H. Berry C. (2020). Treatment of coronary microvascular dysfunction. Cardiovasc. Res. 116 (4), 856–870. 10.1093/cvr/cvaa006 32087007 PMC7061279

[B2] Beer M. Seyfarth T. Sandstede J. Landschütz W. Lipke C. Köstler H. (2002). Absolute concentrations of high-energy phosphate metabolites in normal, hypertrophied, and failing human myocardium measured noninvasively with (31)P-SLOOP magnetic resonance spectroscopy. J. Am. Coll. Cardiol. 40 (7), 1267–1274. 10.1016/s0735-1097(02)02160-5 12383574

[B3] Borlaug B. A. (2020). Evaluation and management of heart failure with preserved ejection fraction. Nat. Rev. Cardiol. 17 (9), 559–573. 10.1038/s41569-020-0363-2 32231333

[B4] Chirinos J. A. Orlenko A. Zhao L. Basso M. D. Cvijic M. E. Li Z. (2020). Multiple plasma biomarkers for risk stratification in patients with heart failure and preserved ejection fraction. J. Am. Coll. Cardiol. 75 (11), 1281–1295. 10.1016/j.jacc.2019.12.069 32192654 PMC7147356

[B5] Cotter G. Voors A. A. Prescott M. F. Felker G. M. Filippatos G. Greenberg B. H. (2015). Growth differentiation factor 15 (GDF-15) in patients admitted for acute heart failure: results from the RELAX-AHF study. Eur. J. heart Fail. 17 (11), 1133–1143. 10.1002/ejhf.331 26333529

[B6] Cunningham J. W. Claggett B. L. O'Meara E. Prescott M. F. Pfeffer M. A. Shah S. J. (2020). Effect of sacubitril/valsartan on biomarkers of extracellular matrix regulation in patients with HFpEF. J. Am. Coll. Cardiol. 76 (5), 503–514. 10.1016/j.jacc.2020.05.072 32731928

[B7] Dunlay S. M. Roger V. L. Redfield M. M. (2017). Epidemiology of heart failure with preserved ejection fraction. Nat. Rev. Cardiol. 14 (10), 591–602. 10.1038/nrcardio.2017.65 28492288

[B8] Fillmore N. Levasseur J. L. Fukushima A. Wagg C. S. Wang W. Dyck J. R. B. (2018). Uncoupling of glycolysis from glucose oxidation accompanies the development of heart failure with preserved ejection fraction. Mol. Med. Camb. Mass. 24 (1), 3. 10.1186/s10020-018-0005-x 30134787 PMC6016884

[B9] Frangogiannis N. G. (2021). Cardiac fibrosis. Cardiovasc. Res. 117 (6), 1450–1488. 10.1093/cvr/cvaa324 33135058 PMC8152700

[B10] Hahn V. S. Yanek L. R. Vaishnav J. Ying W. Vaidya D. Lee Y. Z. J. (2020). Endomyocardial biopsy characterization of heart failure with preserved ejection fraction and prevalence of cardiac amyloidosis. JACC. Heart Fail. 8 (9), 712–724. 10.1016/j.jchf.2020.04.007 32653448 PMC7604801

[B11] Jiang M. Ni J. Cao Y. Xing X. Wu Q. Fan G. (2019). Astragaloside IV attenuates myocardial ischemia-reperfusion injury from oxidative stress by regulating succinate, lysophospholipid metabolism, and ROS scavenging system. Oxidative Med. Cell. Longev. 2019, 9137654. 10.1155/2019/9137654 PMC6612991 31341538

[B12] Kirshenbaum L. A. Singal P. K. (1992). Antioxidant changes in heart hypertrophy: significance during hypoxia-reoxygenation injury. Can. J. physiology Pharmacol. 70 (10), 1330–1335. 10.1139/y92-186 1490252

[B13] Kumar A. A. Kelly D. P. Chirinos J. A. (2019). Mitochondrial dysfunction in heart failure with preserved ejection fraction. Circulation 139 (11), 1435–1450. 10.1161/CIRCULATIONAHA.118.036259 30856000 PMC6414077

[B14] Li L. Wang Y. Guo R. Li S. Ni J. Gao S. (2020). Ginsenoside Rg3-loaded, reactive oxygen species-responsive polymeric nanoparticles for alleviating myocardial ischemia-reperfusion injury. J. Control. release official J. Control. Release Soc. 317, 259–272. 10.1016/j.jconrel.2019.11.032 PMC7384207 31783047

[B15] Liu Q. Docherty J. C. Rendell J. C. Clanachan A. S. Lopaschuk G. D. (2002). High levels of fatty acids delay the recovery of intracellular pH and cardiac efficiency in post-ischemic hearts by inhibiting glucose oxidation. J. Am. Coll. Cardiol. 39 (4), 718–725. 10.1016/s0735-1097(01)01803-4 11849874

[B16] McHugh K. DeVore A. D. Wu J. Matsouaka R. A. Fonarow G. C. Heidenreich P. A. (2019). Heart failure with preserved ejection fraction and diabetes: JACC state-of-the-art review. J. Am. Coll. Cardiol. 73 (5), 602–611. 10.1016/j.jacc.2018.11.033 30732715

[B17] Mishra S. Kass D. A. (2021). Cellular and molecular pathobiology of heart failure with preserved ejection fraction. Nat. Rev. Cardiol. 18 (6), 400–423. 10.1038/s41569-020-00480-6 33432192 PMC8574228

[B18] Ni J. Zhao Y. Su J. Liu Z. Fang S. Li L. (2020). Toddalolactone protects lipopolysaccharide-induced sepsis and attenuates lipopolysaccharide-induced inflammatory response by modulating HMGB1-NF-κB translocation. Front. Pharmacol. 11, 109. 10.3389/fphar.2020.00109 32153412 PMC7047824

[B19] Omote K. Verbrugge F. H. Borlaug B. A. (2022). Heart failure with preserved ejection fraction: mechanisms and treatment strategies. Annu. Rev. Med. 73, 321–337. 10.1146/annurev-med-042220-022745 34379445 PMC9002335

[B20] Paulus W. J. Tschöpe C. (2013). A novel paradigm for heart failure with preserved ejection fraction: comorbidities drive myocardial dysfunction and remodeling through coronary microvascular endothelial inflammation. J. Am. Coll. Cardiol. 62 (4), 263–271. 10.1016/j.jacc.2013.02.092 23684677

[B21] Pellicori P. Zhang J. Cuthbert J. Urbinati A. Shah P. Kazmi S. (2020). High-sensitivity C-reactive protein in chronic heart failure: patient characteristics, phenotypes, and mode of death. Cardiovasc. Res. 116 (1), 91–100. 10.1093/cvr/cvz198 31350553

[B22] Reddy Y. N. V. Rikhi A. Obokata M. Shah S. J. Lewis G. D. AbouEzzedine O. F. (2020). Quality of life in heart failure with preserved ejection fraction: importance of obesity, functional capacity, and physical inactivity. Eur. J. heart Fail. 22 (6), 1009–1018. 10.1002/ejhf.1788 32150314

[B23] Rios F. J. Zou Z. G. Harvey A. P. Harvey K. Y. Nosalski R. Anyfanti P. (2020). Chanzyme TRPM7 protects against cardiovascular inflammation and fibrosis. Cardiovasc. Res. 116 (3), 721–735. 10.1093/cvr/cvz164 31250885 PMC7252442

[B24] Sanders-van Wijk S. van Empel V. Davarzani N. Maeder M. T. Handschin R. Pfisterer M. E. (2015). Circulating biomarkers of distinct pathophysiological pathways in heart failure with preserved vs. reduced left ventricular ejection fraction. Eur. J. heart Fail. 17 (10), 1006–1014. 10.1002/ejhf.414 26472682

[B25] Schiattarella G. G. Altamirano F. Tong D. French K. M. Villalobos E. Kim S. Y. (2019). Nitrosative stress drives heart failure with preserved ejection fraction. Nature 568 (7752), 351–356. 10.1038/s41586-019-1100-z 30971818 PMC6635957

[B26] Schiattarella G. G. Rodolico D. Hill J. A. (2021). Metabolic inflammation in heart failure with preserved ejection fraction. Cardiovasc Res. 117 (2), 423–434. 10.1093/cvr/cvaa217 32666082 PMC8599724

[B27] Tong D. Schiattarella G. G. Jiang N. Altamirano F. Szweda P. A. Elnwasany A. (2021). NAD+ repletion reverses heart failure with preserved ejection fraction. Circ. Res. 128 (11), 1629–1641. 10.1161/CIRCRESAHA.120.317046 33882692 PMC8159891

[B28] Wang Z. Zhu Y. Zhang Y. Zhang J. Ji T. Li W. (2020). Protective effects of AS-IV on diabetic cardiomyopathy by improving myocardial lipid metabolism in rat models of T2DM. Biomed. Pharmacother. 127, 110081. 10.1016/j.biopha.2020.110081 32244194

[B29] Westermann D. Lindner D. Kasner M. Zietsch C. Savvatis K. Escher F. (2011). Cardiac inflammation contributes to changes in the extracellular matrix in patients with heart failure and normal ejection fraction. Circ. Heart Fail. 4 (1), 44–52. 10.1161/CIRCHEARTFAILURE.109.931451 21075869

[B30] Zang Y. Wan J. Zhang Z. Huang S. Liu X. Zhang W. (2020). An updated role of astragaloside IV in heart failure. Biomed. and Pharmacother. 126, 110012. 10.1016/j.biopha.2020.110012 32213428

